# Analysis of factors affecting the technical inefficiency on Indonesian palm oil plantation

**DOI:** 10.1038/s41598-022-07113-7

**Published:** 2022-03-01

**Authors:** Irawati Abdul, Dyah Wulan Sari, Tri Haryanto, Thinzar Win

**Affiliations:** 1 Department of Economics, State of Gorontalo University, Gorontalo, 96128 Indonesia; 2 Department of Economics, Airlangga University, Surabaya, 60285 Indonesia; 3 Department of Economics, Mandalay University, Mandalay, 05032 Myanmar

**Keywords:** Plant evolution, Plant sciences, Environmental social sciences

## Abstract

Indonesia’s palm oil plantation is dominated by three actors. Among three actors, the productivity of smallholder farmers has the lowest productivity. This study aims to analyze the value of technical efficiency and factors affecting the technical inefficiency of palm oil plantations in Indonesia by using the stochastic frontier analysis based on the translog production function. The data used in this study are taken from the Central Statistics Agency (Agricultural Business Household Income Survey) in 2013. The number of samples used was 14,367 farmers. The results revealed that the average value of technical efficiency (58.32%) is still far to reach its optimal, showing that there is still to increase in the efficiency of palm oil plantations in Indonesia. The production function suggests that increasing the number of trees can help to increase the number of outputs. To enhance the technical efficiency, education, age, planting system, seed quality, extension service, and plasma farmer are the significant factors.

## Introduction

Indonesia is the largest palm oil producer and exporter in the world. Indonesian produces up to 56.94% of total world production. Palm oil plays an important role in the national economy in Indonesia because palm oil plantation creates job opportunities, especially in rural areas, provides raw materials for the industrial sector, and also contributes the foreign exchange earnings. In addition, palm oil is the most productive source of vegetable oil for biodiesel. One hectare of palm oil can produce 3.5 tons of vegetable oil. This is much better compared to the second most productive crop, canola, which can only produce 0.8 vegetable oil per hectare of land. Currently, Indonesia is the highest producer of palm oil and ranks number one in the world, while Malaysia is the second-largest producer. The increasing demand for domestic vegetable oil and the large potential for exports of crude palm oil (CPO) have triggered the rapid growth of palm oil plantations in Indonesia. In 2018, palm oil plantation has reached 60% of total plantation and 35.7% of crude palm oil production^1^.

The government responded to the high level of world demand for palm oil from year to year by granting land expansion permits through the Regulation of the Minister of Agriculture of the Republic of Indonesia No. 21 of 2017. This regulation has implications for the addition of increasing land expansion. During the period 2015 to 2019, the land for Indonesia’s palm oil needs to be tended to increase, ranging from 11.26 to 14.6%. In 2015, the area of palm oil land was recorded at 11.75 million hectares and is estimated to increase to 14.59 million hectares in 2019. The production of palm oil (CPO) from 2015 to 2019 has always increased per year. In 2015, the production of palm oil (CPO) was 31.07 million tons, an increase to 42.88 million tons in 2018 or an increase of 20.01%. Meanwhile, in 2019, it is estimated that palm oil (CPO) production will be increased to 48.42 million tons or 5.20%. In 2019, the palm oil plantation increased from 1.88 to 14.60% and the export of palm oil also increased from 26.34 million tons in 2015 to 28.27 million tons in 2019^2^. The main reason for increasing palm oil production is the expansion of land area and it is not because of increase in productivity^3^.

In Indonesia, the palm oil production is dominated by three mains actors, namely, state companies, private companies, and smallholders. The smallholder farmers dominated 41.35% of total palm oil production and their productivity rate is 3.43 ton/hector. The productivity of smallholder farmers is lowest among three actors, showing that they cannot produce their outputs at the best optimal. The low productivity of smallholder farmers showed that they cannot allocate their inputs in efficient ways. The ability of farmers can influence the level of technical efficiency. Smallholder farmers who can use their inputs to achieve the highest outputs can be said to be efficient. So, it is important to analyze technical efficiency and the factors affecting the technical efficiency of smallholder farmers’ plantation of palm oil.

Technical efficiency is influenced by several factors. Varina et al. examined the effects of many factors on the technical efficiency of oil palm plantation, including the age of the farmers, education, extension services^4^. The result showed that age, education and extension service significantly affect the technical efficiency. In the research of technical efficiency of palm oil in Cantral Africa Republic, the education is also an influencing factor to increase the efficiency of farmers^5^. Education and age also significantly contributed the technical efficiency of rubber farmers in Nahon, Thailand^6^.

Ariyanto et al. stated that age and the extension services have strongly effect on the technical efficiency of smallholder farmers in West Kalimantan, Indonesia^7^. Bankole et al. mentioned that the age of the farmer has positive effect on the technical efficiency of smallholder farmers in palm oil plantation in Nigeria^8^. Varina et al. also showed that age, education and extension have a positive effect on technical efficiency of palm oil production^9^. The source of the seed and extension services effect on the technical efficiency of palm oil farmers in Jambi Province in Indonesia with the average efficiency value of 66%, showing that it is still need to improve the efficiency^10^. Age and extension also the efficiency of farmers in Ghana^11^. The technical efficiency is also affected by age of the farmers in Thailand Palm Oil plantation^6^, in East Java, Indonesia^12^ and in Ethopia^13^. Extension service and education have also significantly affected the technical efficiency of farmers^12,14,15^.

According to Schoneveld et al.^16^, there is still a large room to improve the productivity of farmers in palm oil production in Indonesia. Many researchers studied palm oil plantation in Indonesia. Hasnah et al. studied the technical efficiency of palm oil plantations in West Sumatra, finding that the mean technical efficiency value is 66%^17^. Otherwise, the technical efficiency of palm oil cultivation analyzed in Riau province in Indonesia, implying that farmers group, extension program, education level, and farm diversification are the important factors to increase productivity^18^. The independent farmers have higher efficiency value than the partnership farmers and the efficiency value of 0.73 also showed that there is still need to fill the efficiency gap in the West Sumatera Province in Indonesia^19^. Ismiasih examined the technical efficiency of palm oil plantation in West Kalimantan and the result showed that the value of technical efficiency is moderate and still need to improve the efficiency^20^. Fariani et al. analyzed the technical efficiency and factor affecting the technical efficiency in South Sumatra^21^. However, studies measuring the technical efficiency by using five inputs and determinants of technical inefficiency using the country-level data are still limited. We aim to fill this research gap. This paper aims to analyze the technical efficiency and factors affecting the technical inefficiency of palm oil plantations in Indonesia using the stochastic frontier approach (SFA). We cover a sample of 14,367 firms in Indonesia in 2013.

This paper proceeds as follows material and method in “Materials and methods” section, results and discussion presented in “Results and discussion” section, and the end of research in last section.

## Materials and methods

### Area descriptions

The data in this study are taken from the official secondary data released by the Central Bureau of Statistics (BPS) in Indonesia through the Agriculture Survey (Agriculture Household Income Survey) data for Indonesia in 2013. The Central Bureau of Statistics in Indonesia conducted the Agriculture Survey data every 10 years. So, this data set is the latest survey data set conducted by the Central Bureau of Statistics and covered the data for country-level data. The samples were 14,367 firms.

### Stochastic frontier analysis

In this study, we used the stochastic frontier analysis in which the efficiency frontier is based on econometric modeling. Aigner et al. and Meeusen et al. established the stochastic frontier production function^22,23^. This function includes two kinds of disturbance terms: one is for inefficiency term and the other permits random errors to affect production. The stochastic frontier production function has two principal merits because of dealing with stochastic noise and permitting the statistical tests of hypotheses with the structure of production and inefficiency value^24^.

In numerous pieces of literature, the determinants of inefficiency can be accessed by utilizing a two-stage estimation process. In stage one, *u*_*i*_ can be accessed from the stochastic frontier production function. In stage two, values of *u*_*i*_ gained from the first stage are regressed against firm-specific variables that are expected to clarify the distinctions *u*_*i*_ between firms. Battese and Coelli demonstrated that these firm-specific factors should be simultaneously estimated in the production frontier estimation because these variables may directly affect the inefficiency^25^. In this study, the parameters of the stochastic production frontier and the inefficiency model are simultaneously estimated. This model can be composed as Eq. (1):

1 $${\text{Y}}_{\text{i}}\,{=}\,{\text{X}}_{\text{i}}{\upbeta+}\left({\text{v}}_{\text{i}}-{\text{u}}_{\text{i}}\right), \quad \text{i} =1, \ldots, \text{N,}$$

where Y_i_ = the production of the i-th firm; X_i_ = a k × 1 vector of inputs quantities of the i-th firm; β = a vector of unknown parameters; v_i_ = random variables which are thought to be independent and identically distributed N (0, σ_v_^2^) and independent of the u_i_ which are non-negative random variables which are expected to represent the inefficiency term in production and are assumed to be independently distributed as truncations at zero of the N(m_i_,${\sigma }_{u}^{2}$) distribution and can be shown by Eq. (2)

2 $${\text{m}}_{{\text{i}}} = {\text{ z}}_{{\text{i}}} \delta ,$$

where z_i_ = a p × 1 vector of variables which may affect the efficiency of a firm, δ = a 1 × p vector of the parameter to be estimated.

The parameterization used in this model is ${\sigma}_{\text{u}}^{2}$ and ${\sigma}_{\text{v}}^{2}$ with ${\sigma}^{2}= \text{ } {\sigma}_{\text{v}}^{2} + {\sigma}_{\text{u}}^{2}$ and ${\upgamma} = {{\sigma}_{\text{u}}^{2}}{/ (}{{\sigma}_{\text{v}}^{2}} {+} {{\sigma}_{\text{u}}^{2}}\text{)}$.

The stochastic production model can be expressed in Eq. (3) by following Battese and Coelli^25^,

3 $${lny}_{i}={\beta }_{0}+\sum_{m}{\beta }_{m}ln{x}_{mi}+{\varepsilon }_{i},$$

where, y represents the output, x represents the inputs used in the production and $\beta$ is the coefficients. From Eq. (3), the technical efficiency of palm oil can be calculated by using Eq. (4). The technical efficiency (TE_i_) is measured by calculating it from the ratio of the observed output to the maximum output (frontier).

4 $${TE}_{i}=\frac{{y}_{i}}{{y}_{i}^{*}}=\frac{\mathrm{exp}({x}_{i}\beta +{v}_{i}-{u}_{i})}{\mathrm{exp}({x}_{i}\beta +{v}_{i})}=e\mathrm{xp}\left(-{u}_{i}\right).$$

The value of technical efficiency is between one and zero ($$0<{TE}_{i}<1)$$. The more the value of technical efficiency is near one, the more the firm is efficient.

Following Battese and Coelli^25^, the ${u}_{i}$ are supposed non-negative random variables and this characterize the stochastic deficiency of outputs from the most efficient production. It is supposed that ${u}_{i}$ is characterized by truncation of the normal distribution with mean and can be presented as Eq. (5):

5 $${\mu}_{\text{i }}= {\delta }_{0}\text{+}\sum_{\text{j=1}}^{\text{J}}{\updelta}_{\text{j }}{\text{z}}_{\text{ji}},$$

and variance, ${\sigma }^{2}$, where ${z}_{ji}$ is the estimation of j-th explanatory related to the technical inefficiency effect of the firm i and ${\delta }_{0}$ and ${\delta }_{j}$ are unknown parameters to be estimated. Here, ${z}_{ji}$ is the vector of elements that may be associated with inefficiency and which may fluctuate over time. The inefficiency model’s random component is not identically distributed nor is it needed to be nonnegative^24^.

In this study, the production function is based on trans-log form and the Eq. (3) can be expanded as Eq. (6),

6 $${lnY}_{i}={\beta }_{0}+{\beta }_{po}{lnPo}_{i}+{\beta }_{pu}{lnPu}_{i}+{\beta }_{Ps}{lnPs}_{i}+{\beta }_{Tk}{lnTk}_{i}+{\beta }_{ln}{lnLa}_{i}+0.5{\beta }_{Po}({lnPo}_{i}{)}^{2}+0.5{\beta }_{Pu}({lnPu}_{i}{)}^{2}+0.5{\beta }_{Ps}({lnPs}_{i}{)}^{2}+0.5{\beta }_{Tk}({lnTk}_{i}{)}^{2}+0.5{\beta }_{La}({lnLa}_{i}{)}^{2}+{\beta }_{Po}{\beta }_{Pu}{lnPo}_{i}{lnPu}_{i}+{\beta }_{Po}{\beta }_{Ps}{lnPo}_{i}{lnPs}_{i}+{\beta }_{Po}{\beta }_{Tk}{lnPo}_{i}{lnTk}_{i}+{\beta }_{Po}{\beta }_{La}{lnPo}_{i}{lnLa}_{i}+{\beta }_{Pu}{\beta }_{Ps}{lnPu}_{i}{lnPs}_{i}+{\beta }_{Pu}{\beta }_{Tk}{lnPu}_{i}{lnTk}_{i}+{\beta }_{Pu}{\beta }_{La}{lnPu}_{i}{lnLa}_{i}+{\beta }_{Ps}{\beta }_{Tk}{lnPs}_{i}{lnTk}_{i}+{\beta }_{Ps}{\beta }_{La}{lnPs}_{i}{lnLa}_{i}+{\beta }_{Tk}{\beta }_{La}{lnTk}_{i}{lnLa}_{i}+{v}_{i}+{u}_{i},$$

where, Y_i_ represents the output of palm oil plantations on the ith plantation, the inputs used in the estimation process were Po (trees), Pu (fertilizer), Ps (pesticides), Tk (labor), and La (land), where β_i_ is the estimated coefficient. The determinants of technical inefficiency are shown in Eq. (7).

7 $${u}_{i}={\delta }_{0}+{\delta }_{1}{pen}_{i}+{\delta }_{2}{um}_{i}+{\delta }_{3}{sp}_{i}+{\delta }_{4}{kb}_{i}+{\delta }_{5}{opt}_{i}+{\delta }_{6}{peny}_{i}+{\delta }_{7}{cr}_{i}+{\delta }_{8}{ass}_{i}+{\delta }_{9}{plas}_{i}+{\varepsilon }_{i},$$

where, ${u}_{i}$= technical inefficiency; $\delta$ = coefficients; pen = Education level of farmer; um = age of the farmer; sp = planting system; kb = Seed quality; opt = Pests; peny = extension services cr = credit; ass = member of farmer association and plas = plasma farmer.

### Data and variables

Palm oil output (y) is calculated based on the production results including all the value of the primary product, the value of by-products, the value of self-harvested production, and the value of slash, and it is measured by thousand rupiahs. Trees (po) are calculated as a weighted tree (WT) to capture the age of palm oil trees^16,17,24^. There are two methods to calculate the WT: (1) estimating the data by using the non-linear least square regression and (2) calculating the WT by using the two-age yield profiles from the literature^17^. This study used the second method by following Varina, Hartoyo, Kusnadi and Rifin^21^. The weighted trees can be defined as:

8 $${WT}_{i}={k}_{1}{WT}_{1i}+{k}_{2}{WT}_{2i}+{k}_{3}{WT}_{3i},$$

where ${WT}_{i}$ is the weighted numbers of trees on farmer i. ${WT}_{1}$, ${WT}_{2}$, ${WT}_{3}$ are the age categories of 3–7 years, 8–16 years, and above 16 years respectively. ${k}_{1}$, ${k}_{2}$ and ${k}_{3}$ are the weight of each age category. According to Varina et al.^4^, the value of ${k}_{1}$, ${k}_{2}$ and ${k}_{3}$ are 0.81, 1 and 0.98 respectively.

Fertilizer (pu) includes several types of fertilizers, namely Urea, TSP/SP36, ZA, KCL, NPK, organic fertilizers (manure/compost), and other fertilizers and are expressed in thousand rupiahs. Pesticides (ps) include solid and liquid pesticides which are measured in thousand rupiahs. Labor (tk) is the number of people employed in palm oil plantations. Land area (la) is not expressed in the census data so that the researcher calculated the land area by multiplying the number of trees with the spacing between trees. The education (pen) is based on the education level of the farmers and is measured by the year of schooling. Age (um) is the age of the farmer. Planting systems (sp) are expressed in dummy variables and if the farmers use a single planting system (1) and otherwise (0).

Seed quality (kb) is divided into the uncertified seed (0) and certified seed (1). Pests (Opt) are the pests, weed diseases, and others that are exposed to the palm oil product during the planting period, and the trees are exposed to pests (1) and otherwise (0). Extension service is whether members of the farmer in one household receive counseling (1) about the management of palm oil plantations or do not receive counseling (0). The farmers have credit access (1) and otherwise (0). Farmers are a member of farmer association (1) and otherwise (0). Farmers are plasma farmers (1) and otherwise (0). Table 1 shows the descriptive analysis of palm oil production.

**Table 1:** Descriptive statistics.

Variabel	Unit	Obs	Mean	Std. Dev.	Min	Max
y (output)	Thousand rupiah	14,367	47,098.41	646.29	33	2,235,600
po (tree)	Number of weighted tree	14,367	295.81	4.19	2	24,300
pu (fertilizer)	Thousand rupiah	14,367	5629.57	108.49	5	474,400
ps (pesticide)	Thousand rupiah	14,367	1000.95	24.79	2	145,000
tk (labor)	Number of people	14,367	3.16	0.04	1	180
la (land)	Hectar	14,367	4.39	0.27	0.001	2160
pen (education)	Years of schooling	14,367	2.51	0.01	0	14
um (age)	Year	14,367	47.056	0.09	17	99
sp (planting system)	Dummy	14,367	0.97	0.001	0	1
kb (seed quality)	Dummy	14,367	0.49	0.004	0	1
opt (pest)	Dummy	14,367	0.68	0.004	0	1
peny (extension services)	Dummy	14,367	0.10	0.002	0	1
cr (credit)	Dummy	14,367	0.14	0.003	0	1
ass (member of farmer association)	Dummy	14,367	0.01	0.001	0	1
plas (plasma farmer)	Dummy	14,367	0.17	0.003	0	1

## Results and discussion

Before analyzing the results, this study made the hypothesis testing to choose the best functional form for this data set by using the likelihood ratio (LR) test. Firstly, it is tested for choosing the best model. According to the results, the null hypothesis is rejected. Thus, the translog model is chosen for the data analysis. The LR test is shown in Table 2.

**Table 2:** Hypothesis testing.

Model	H_0_	$\lambda$	χ^2^ (at 1%)	Conclusion
Cobb–Douglass	${\beta }_{lala}={\beta }_{PoLa}=0$	128.9	15.08	Reject *H*_0_

The value of $\lambda$ is calculated from LR test.

Table 3 shows that the estimated results of nine variables that are affecting the technical inefficiency. Among nine variables, seven variables affect technical inefficiency, namely, education, age, planting system, seed quality, pests, extension services, and plasma farmers. In inefficiency model, the negative sign of variable shows an increase in efficiency and the positive sign shows an decrease in inefficiency^25^.

**Table 3:** Maximum likelihood estimation of palm oil production.

Variable	Coefficient	Estimated value	Standard error
Constant	${\beta }_{0}$	5.6014***	0.1760
*Po*	${\beta }_{po}$	0.6134***	0.0523
*Pu*	${\beta }_{pu}$	0.1388***	0.0417
*Ps*	${\beta }_{ps}$	0.0309	0.0436
*tk*	${\beta }_{tk}$	0.2691***	0.0651
*La*	${\beta }_{la}$	0.0637***	0.0310
*po*^*2*^	${\beta }_{popo}$	− 0.0956***	0.0514
*pu*^*2*^	${\beta }_{pupu}$	0.0288***	0.0067
*ps*^*2*^	${\beta }_{psps}$	− 0.0226***	0.0069
*tk*^*2*^	${\beta }_{tk}$	− 0.0534***	0.0164
*la*^*2*^	${\beta }_{lala}$	− 0.0062**	0.0031
*Popu*	${\beta }_{popu}$	0.0159*	0.0085
*Pops*	${\beta }_{pops}$	0.0662**	0.0094
*Pol*	${\beta }_{pol}$	0.0340**	0.0143
*Pola*	${\beta }_{pola}$	− 0.0094	0.0066
*Pups*	${\beta }_{pups}$	− 0.0288***	0.0061
*Putk*	${\beta }_{putk}$	− 0.0535***	0.0105
*Pula*	${\beta }_{pula}$	− 0.0056	0.0044
*Pstk*	${\beta }_{psl}$	0.0175	0.0116
*Psla*	${\beta }_{psla}$	0.0033	0.0053
*tkla*	${\beta }_{tkla}$	0.0165**	0.0081
**Inefficiency model**			
Constant	${\updelta }_{0}$	− 7.3049***	0.6918
Education	${\updelta }_{1}$	− 0.2027***	0.0168
Age	${\updelta }_{2}$	− 0.0684***	0.0035
Planting system	${\updelta }_{3}$	− 5.7006***	0.6254
Seed quality	${\updelta }_{4}$	− 0.5898***	0.0519
Pests	${\updelta }_{5}$	0.7953***	0.0499
Extension services	${\updelta }_{6}$	− 2.1995***	0.1811
Credit	$7$	− 0.0465	0.0828
Member of farmer association	${\updelta }_{8}$	0.2115	0.5965
Plasma farmer	${\updelta }_{9}$	− 6.7274***	0.1428
Sigma Square	${\upsigma }^{2}$	11.3794***	0.5027
Gamma	$\upgamma$	0.9837***	0.0007
Log-likelihood function		− 15,070.10	
Error one-sided LR test		3023.9	

***, ** and ** are the significant level of 10%, 5% and 1% respectively.

Education has a negative and significant coefficient of technical inefficiency which implies that the level of education can increase the technical efficiency of palm oil plantations. The more the farmers are educated, the more the technical efficiency. This is also in line with the finding of Alwarritzi and Varani et al., in which the higher the education level of palm oil farmers, the more responsive they tend to be in adopting and utilizing palm oil management technology^9,21^. Fariani et al. and Varani et al. also confirmed that the higher the level of farmer education reduces the level of technical inefficiency and the education increases the level of efficiency^9,21^. This is because Indonesia government encourage a social intervention such as Corporate Social Responsibility (CSR) in the palm oil plantation in Indonesia. CSR provide training and education program for farmers, there by improve the efficiency of palm oil plantation^26^. This also in line with the policy of government program via Ministry of Agriculture to empower the farmers capacity in training and education practices specially how to increase the quality of plantation palm oil. The coefficient of farmers’ age is negative and significant. This means that the older the age of the farmers, the more efficiency in palm oil plantation. This is because the older farmers have more experience than younger farmers and they can improve the efficiency according to their experience. This finding is consistent with Hasnah et al., Fariani et al. and Varina^9,17,21^. The planting system has a negative and significant value on technical inefficiency. This means that the farmer who uses the single systems will increase the level of technical efficiency.

The estimation result shows that the quality of the seeds has a negative effect on technical inefficiency. This means that ‘certified’ seeds can increase technical efficiency compared to ‘uncertified’ seeds. This is confirmed by the results of research conducted by Kariyasa in which found that certified seeds significantly increased the productivity level of palm oil by 66.34% more compared to uncertified seeds^27^. This also have already done by government of Indonesia via Ministry of agriculture program at Research and Development Plantation to develop research specially in producing the quality of seed with resilience and adapted to climate. Pests have a positive and significant value for technical inefficiency. This means that most palm oil farmers who are ‘not exposed’ to plant-disturbing organisms are technically more efficient than those who are ‘exposed’ to plant-disturbing organisms.

Extension service has a significant effect with negative value on technical inefficiency. This shows that the majority of palm oil farmers receive counseling and they tended to have a higher value of technical efficiency than the farmers who do not receive counseling. This is constituent with the finding of Alwarritzi et al.^18^ and Varina et al.^4,8^. Related with the research above, the Government has been improving the program, that is offered the extension service to follow the training and education specially how they use the technology communication system so the target of extension service in transforming the skill and expertise to the farmer will be more easily and effecting to be implementing, so this also as a part of policy. The coefficient of plasma farmers has a negative value and significant in technical inefficiency, showing that the plasma farmers are more efficient than non-plasma farmers. This is because the plasma farmers can get more facilities by involving the plasma farmer group and they can get fully guidelines supported by their contact company. This result is in line with the findings of Ismiasih^20^ and Alwarritzi et al.^18^.

Table 4 shows the output elasticity of each input variable such as trees (po), fertilizer (pu), pesticides (ps), labor (tk), and land area (la). Output elasticity is obtained by partially taking the first-order derivative of the selected translog model. Furthermore, each variable will be calculated at the average value of the entire sample data. Output elasticity means how much the percentage of output will increase when the input is increased. In this study, all the elasticity of input variables is positive and inelastic. This shows that an increase of 1% use in every input will cause an increase in the output of less than 1% in this production function. The total value of elasticity (1.0243) is greater than one, meaning that the palm oil production has an increasing return to scale.

**Table 4:** Estimation results of output elasticity concerning each input.

Variables	Elasticity
Elasticity of tree (${\varepsilon }_{po}$)	0.6784
Elasticity of fertilizer $({\varepsilon }_{pu}$)	0.2161
Elasticity of pesticide $({\varepsilon }_{ps}$)	0.0306
Elasticity of Labor $({\varepsilon }_{tk}$)	0.0947
Elasticity of land $({\varepsilon }_{la}$)	0.0062
Total (ε)	1.0260

Total elasticity = $${\varepsilon }_{po}+{\varepsilon }_{pu}+{\varepsilon }_{ps}+{\varepsilon }_{tk}+{\varepsilon }_{la}$$.

The average elasticity of the tree on palm oil output is the highest elasticity value of 0.6784. This shows that an increase in output produced by palm oil plantations is due to an increase in the number of trees. Furthermore, the elasticity of the fertilizer variable has the second-largest elasticity value after the tree variable with a value of 0.2161, meaning that the farmers need to use the fertilizer to increase the output. The elasticity of the pesticide variable is 0.0306, meaning that an increase in palm oil output by 1unit requires fertilizer of 0.0306 because the types of palm oil plants are hardy and after the age of 10 years, the palm oil plants do not require large amounts of pesticides. The value of the elasticity of labor is 0.0947, meaning that the plantation of palm oil in Indonesia still uses the traditional method and still needs to increase the labor. The elasticity of the land variable on the output has a value of 0.0062, meaning that an increase in the amount of land on palm oil plantations increases the output.

The mean value of technical efficiency is shown in Table 5. According to the results, the value of technical efficiency is relatively inefficient. The average value of technical efficiency is 0.5832. This shows that there is room to increase the productivity and efficiency of palm oil production. The results of the mean value of technical efficiency in each province show that there were three regions with the highest technical efficiency values, namely West Sumatera (0.6956), North Sumatra (0.6707), and West Kalimantan (0.6670). Banten province is the lowest efficiency value.

**Table 5:** Mean value of palm oil technical efficiency.

No	Province	Technical efficiency
1	West Sumatera	0.6956
2	North Sumatera	0.6707
3	West Kalimantan	0.6670
4	West Sulawesi	0.6503
5	Bengkulu	0.6429
6	South Sumatera	0.6402
7	Riau	0.6391
8	Jambi	0.6276
9	East Kalimantan	0.6261
10	Central Kalimantan	0.5905
11	Aceh	0.5885
12	South Kalimantan	0.5829
13	Lampung	0.5565
14	Central Sulawesi	0.5414
15	Kepulauan Bangka Belitung	0.5079
16	Southeast Sulawesi	0.4996
17	South Sulawesi	0.4546
18	Banten	0.3163
Average		0.5832

**Figure 1:**
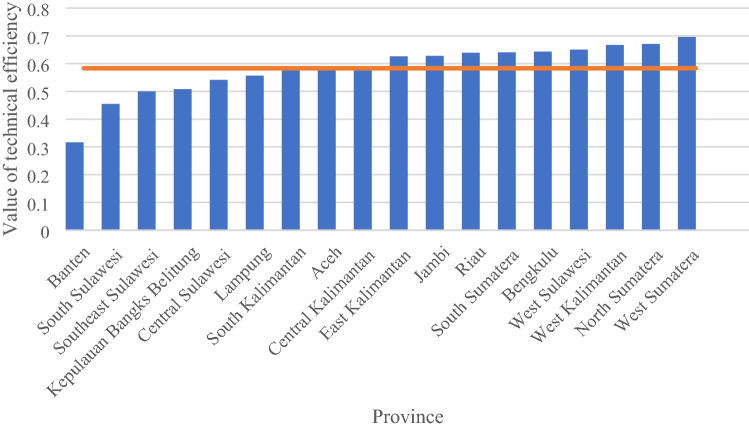
Mean technical efficiency of palm oil plantation.

In Fig. 1, it can be seen that, the value of technical efficiency in each province shows that there are eight regions that are below the average the technical efficiency value of 0.5832. This value (0.5832) meaning that the smallholder farmers need to improve 42% of their efficiency to follow the best practice benchmark value of 1. This is also in line with the literature of Woittiez et al.^28^.

**Figure 2:**
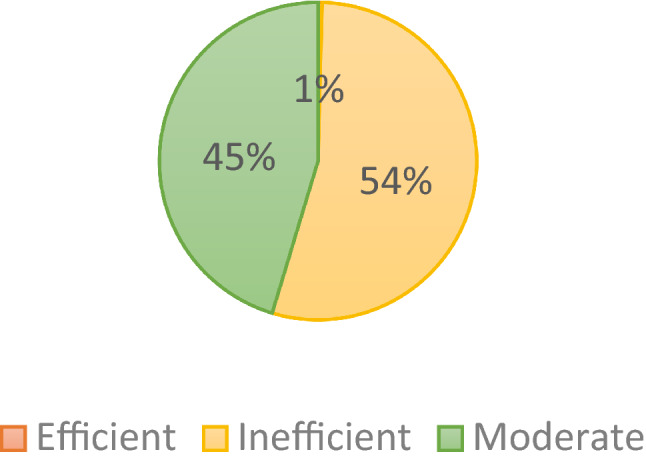
Distribution of technical efficiency.

Figure 2 shows that the distribution of the level of technical efficiency of farmers. If the value of technical efficiency is greater than equal to 9, this firm is regarded as an efficient firm. The firm with moderate efficiency has the efficiency value of above 0.7 and below 0.9. The firm is assumed as inefficient if its efficiency value is less than 0.7^5^. In Indonesia, only 1% of the farmers are efficient in their production. It can be said that the palm oil production in Indonesia need to increase their resources to improve their efficiency. 54% of total farmers owns the moderate efficient value and 45% of farmers are working with low efficiency.

## Conclusions

In Indonesia, palm oil farmers face technical inefficiencies. The estimation results showed that the technical efficiency of palm oil plantations has an average value of 0.6257. This showed that technical efficiency of smallholder farmers is still needed to improve. Among the inputs, number of trees has the highest effect on the output of palm oil. By increasing the number of trees, the output of palm oil can be increased. Thus, the government should create land opportunities to increase the plantation of palm oil. According to the estimated results, education, age, planting system, seed quality, extension services, and plasma farmer are important factors for improving the technical efficiency. Improving the education level of farmers can increase the knowledge on technology relating to agricultural practices, thereby can increase efficiency. Thus, government should support the training and should motivate the educated people to include the oil palm plantation. For the planting system, farmer should be practicing the single farming system to improve the technical efficiency. Using the good quality seed can improve the technical efficiency and the government should encourage the farmers to use certified seeds and seek new methods to get more qualified certified seeds. Extension service is a very important factor because farmers can get the systematic methods for their plantation from the extension service. The government should increase the officers for extension service to improve the technical efficiency of smallholder farmers.
